# The causal effects of education on adult health, mortality and income: evidence from Mendelian randomization and the raising of the school leaving age

**DOI:** 10.1093/ije/dyad104

**Published:** 2023-07-18

**Authors:** Neil M Davies, Matt Dickson, George Davey Smith, Frank Windmeijer, Gerard J van den Berg

**Affiliations:** Division of Psychiatry, University College London, London, UK; Department of Statistical Sciences, University College London, London, UK; K.G. Jebsen Center for Genetic Epidemiology, Department of Public Health and Nursing, Norwegian University of Science and Technology, Tronheim, Norway; Medical Research Council Integrative Epidemiology Unit, University of Bristol, Bristol, UK; Institute for Policy Research, University of Bath, Bath, UK; Medical Research Council Integrative Epidemiology Unit, University of Bristol, Bristol, UK; Population Health Sciences, University of Bristol, Bristol, UK; Medical Research Council Integrative Epidemiology Unit, University of Bristol, Bristol, UK; Department of Statistics and Nuffield College, University of Oxford, Oxford, UK; Department of Economics, University of Groningen, Groningen, The Netherlands; Department of Epidemiology, University Medical Center Groningen, Groningen, The Netherlands

**Keywords:** Raising Of School Leaving Age (ROSLA), instrumental variable analysis, education, genomic confounding

## Abstract

**Background:**

On average, educated people are healthier, wealthier and have higher life expectancy than those with less education. Numerous studies have attempted to determine whether education causes differences in later health outcomes or whether another factor ultimately causes differences in education and subsequent outcomes. Previous studies have used a range of natural experiments to provide causal evidence. Here we compare two natural experiments: a policy reform, raising the school leaving age in the UK in 1972; and Mendelian randomization.

**Methods:**

We used data from 334 974 participants of the UK Biobank, sampled between 2006 and 2010. We estimated the effect of an additional year of education on 25 outcomes, including mortality, measures of morbidity and health, ageing and income, using multivariable adjustment, the policy reform and Mendelian randomization. We used a range of sensitivity analyses and specification tests to assess the plausibility of each method’s assumptions.

**Results:**

The three different estimates of the effects of educational attainment were largely consistent in direction for diabetes, stroke and heart attack, mortality, smoking, income, grip strength, height, body mass index (BMI), intelligence, alcohol consumption and sedentary behaviour. However, there was evidence that education reduced rates of moderate exercise and increased alcohol consumption. Our sensitivity analyses suggest that confounding by genotypic or phenotypic confounders or specific forms of pleiotropy are unlikely to explain our results.

**Conclusions:**

Previous studies have suggested that the differences in outcomes associated with education may be due to confounding. However, the two independent sources of exogenous variation we exploit largely imply consistent causal effects of education on outcomes later in life.

Key Messages

- On average, more educated people are healthier and more long-lived.
- We do not know whether these differences are caused by educational attainment or other factors affecting education and health.
- We compared estimates of the effects of education derived from multivariable adjustment and two natural experiments: the first, an educational policy reform; and the second, the random inheritance of DNA from parents to offspring.
- The estimates of the effects of educational attainment suggested that educational attainment leads to better health and social outcomes.

## Introduction

Educational decisions such as remaining in school, made comparatively early in life, associate with substantial differences in outcomes across the life course.^1–7^ Unfortunately for researchers interested in the causal effects of education, these decisions do not occur randomly. For example on average, people who remain in school for longer are more likely to have educated parents. Thus it is challenging to determine if education causes differences in outcomes later in life or if other, potentially unknown, factors drive these associations. As a result, approaches such as multivariable adjustment may suffer from residual confounding.^8^ Instrumental variable analysis can provide an alternative source of evidence about the causal effects of education and may be unbiased, even given unmeasured confounding of the education-outcome association. Three assumptions define instrumental variables: (i) they must associate with the risk factor of interest (the ‘relevance/informativeness criterion’); (ii) they have no common cause with the outcome (‘the independence assumption’); and (iii) they do not affect the outcome except via the risk factor of interest (the ‘exclusion restriction’).^9^

Natural experiments, such as legal changes to raise the school leaving age, are potential instrumental variables for educational attainment. These changes forced people to remain in school for longer and, because parents could not have anticipated them, are unlikely to be associated with factors that confound the association between education and other outcomes. The effect of additional years of education can be estimated using instrumental variable estimators.^1^

Another potential source of instrumental variables is Mendelian randomization, involving genetic variants that are known to associate with educational attainment.^5^^,^^10^^,^^11^ This approach exploits the natural experiment at conception—when each child inherits half of their parents’ genomes. Specifically, there is a 50% chance of inheriting one or other of their parents’ alleles at each locus. The first instrumental variable assumption is likely to hold because large genome-wide association studies (GWAS) have discovered genetic variants robustly associated with education. Because of the segregation of alleles at conception, these genetic variants may be independent of many confounders and genetic variants for other traits like cardiovascular disease. On average, phenotypes tend to cluster and be more associated with each other than expected by chance, whereas genetic variants known to associate with one trait tend to be independent of other potential risk factors.^12^ Furthermore, germline DNA is fixed at conception and cannot be affected by later educational attainment or other outcomes. Thus, reverse causation from health or socioeconomic outcomes to germline DNA is impossible. Genetic variants have been used as instrumental variables for educational attainment. Still, few studies have directly compared estimates from policy reforms and genetic instrumental variables (see Supplementary Table S1, available as Supplementary data at *IJE* online for previous Mendelian randomization studies of educational attainment).

Here we compare two potential instrumental variables, a policy reform and Mendelian randomization, within the same sample. We have previously reported the effects of educational attainment using the raising of the mandatory minimum school leaving age, using data from the UK Biobank.^13^ We assess the plausibility of the Mendelian randomization assumptions for estimating the effects of educational attainment. We estimate the long-term effects of education, using both genetic variants and the raising of the school-leaving age.

## Methods

### Data

We used data from the UK Biobank, which sampled and obtained consent from 503 317 people via 23 study centres in urban areas across the UK. The study invited people aged between 40 and 70 to attend an assessment clinic between 2006 and 2010. The participants completed surveys, had detailed phenotypic measurements and provided blood samples. See Supplementary Material Section 2.1 (available as Supplementary data at *IJE* online) for details of genotyping quality control.

### Educational attainment

For the observational and Mendelian randomization analyses, we used years of schooling as the exposure; for the raising of the school leaving age, we used whether someone had remained in school after age 15. Within the instrumental variables framework, both exposures indicate the effect of an additional year of schooling. We derived each participant’s years of schooling using the information they provided when using a touch screen survey as part of their assessment centre visit. This survey included questions on the participant's educational qualifications (i.e. whether they had a degree or A levels). We used these variables to measure educational attainment based on the International Standard Classification of Education (ISCED). Okbay and colleagues used this definition; we recoded the number of years of education for each category referred to, to be consistent with the UK education system.^14^ See the Supplementary Material (available as Supplementary data at *IJE* online) for detailed coding. If the participant stated that they did not have a degree, they were asked at what age they left school. We used these survey responses to determine whether the participant remained in school after age 15. If the participant did not have a degree, then this was equal to one if they stated they left school after the age of 15. Otherwise, it was set equal to zero. If they said they had a degree, this variable was set to one.

### Outcomes

#### Morbidity

The participants completed questionnaires about whether a doctor had diagnosed them with high blood pressure, stroke or heart attack. They were asked if they had been diagnosed with diabetes. We set this outcome to missing if they received a diagnosis before age 21. They were also asked if they had experienced episodes of depression. Finally, cancer diagnoses were defined using linked cancer registry data.

#### Mortality

Mortality was defined using linked NHS mortality records. This dataset included the date of death for all participants which had occurred after attending the clinic until the 17 February 2014.

#### Health behaviours

The participants were asked detailed questions about their smoking history. From this, information was derived about whether they currently or had ever smoked. They were asked about their alcohol consumption, coded as an ordinal variable (0 = never, 1 = special occasions only, 2 = one to three times a month, 3 = once or twice a week, 4 = three or four times a week, and 5 = daily or almost daily). They were asked how many hours they spent watching television per day and how many days per week they did 10 min or more of moderate or vigorous physical activity.

#### Income

The participants were asked about their average, total, before-tax, annual household income. We transformed this into four binary variables indicating whether their income was above £18 000, £31 000, £52 000 or £100 000.

#### Indicators of ageing

During the clinic visit, grip strength measures were taken on both hands using a Jamar J00105 hydraulic hand dynamometer. The measurements for each hand were averaged and residualized to account for between-device differences, accounting for 2.92% of the variation in grip strength. Pulse-wave arterial stiffness was measured from the finger using an infra-red sensor (PulseTrace PCA2, CareFusion, USA). These measurements were residualized to account for between-device differences, accounting for 2.53% of the variation in arterial stiffness.

#### Anthropometry

We derived height and body mass index (BMI) using the participant’s standing height (measured using a Seca 202 measuring rod) and their weight.

#### Blood pressure

The participant's blood pressure was measured twice using an Omron 705 IT electronic blood pressure monitor. These measurements were averaged to calculate diastolic and systolic blood pressure.

#### Neurocognitive

The participants used a touchscreen to complete a battery of 13 fluid intelligence questions. The participants were given 2 min to answer as many questions correctly as possible. The participants were also asked if they were extremely, very or moderately happy or unhappy.

### Statistical methods

We investigated two potential instrumental variables for educational attainment: (i) the raising of the school leaving age in 1972; and (ii) an allele score (called the ‘educational attainment genetic score’) constructed using results from the discovery sample of a GWAS of years of education in an independent sample.^14^

#### The raising of the school leaving age

In September 1972, the minimum school leaving age in the UK increased from 15 to 16. This forced participants who would otherwise have left school to remain in school for an extra year. Many studies have used this policy reform to estimate the effect of schooling on later outcomes. It is a plausible natural experiment because parents of children affected by the reform could not have anticipated the change in the law at the time of conception. So on average, individuals affected by the reform will be similar to those who were not affected. We used a 12-month bandwidth. This analysis compares the outcomes of the individuals in the first school cohort affected by the reform with the outcomes of the last cohort who were not affected. We accounted for linear secular trends in the outcome using a difference in difference design. We subtracted the average year-on-year difference for the cohorts born in the 10 years before and after the reform; see Davies N *et al*. for further details.^13^

#### Allele scores for educational attainment

We constructed the allele scores using the 74 single nucleotide polymorphisms (SNPs) associated with years of education (*p *<5 × 10^–08^) in the discovery sample of Okbay and colleagues.^14^ We did not use more recent GWAS of educational attainment because they contain the UK Biobank sample and we wanted to minimixe sample overlap.^15^^,^^16^ Five SNPs reported by the GWAS were not available in the Haplotype Reference Consortium (HRC) panel; we replaced these SNPs with proxies that were in perfect linkage disequilibrium (LD) and the HRC panel. See Supplementary Table S2 (available as Supplementary data at *IJE* online) for a list of the specific GWAS used. The allele scores are the weighted sum of the number of education-increasing alleles for each participant. The contribution of each SNP to the score was weighted by the size of the coefficient reported by the GWAS. The effect alleles of all GWAS results were harmonized to be consistent with the UK Biobank genome-wide data. We excluded palindromic SNPs with a minor allele frequency of 0.5 or above. We checked for consistency between the effect allele frequency between the GWAS and UK Biobank data. The allele frequencies were highly correlated, ρ= 0.9968, and the maximum difference in allele frequency of the SNPs was 0.048.

### Specification tests

Recall that three assumptions define instrumental variables: (i) they must be associated with the risk factor of interest; (ii) they must have no common cause with the outcome (no confounding); and (iii) they must have no direct effect on the outcome (the exclusion restriction). We tested whether the first assumption held using a partial F statistic of the instrument-exposure association. We investigated the plausibility of the second assumption by estimating the association of each instrument and a broad set of phenotypic and genetic confounders (defined below). We used covariate balance plots to account for the relative strength of the instruments.^17^ Covariate balance plots plot the ratios of the instrument's association with the measured confounders and the the instrument's associationwith the exposure (educational attainment).^18^ We estimated these terms using the generalized method of moments.^19^

### Sample selection

The UK Biobank is a highly non-random sample that oversampled those with degrees and sampled relatively few people with little education or no qualifications. This sampling method could cause collider bias in our sample if the sample selection relates to both the outcome and the potential instruments. We accounted for this non-random sampling using inverse probability weights. Individuals who reported leaving school at age 15 were weighted by 34.29, and the selected participants who left school at age 16 or older were weighted by 12.37 (weights rounded to two decimal places).^20^ We investigated whether our results were sensitive to the specification of these weights in a sensitivity analysis in the Supplementary Material; see Hughes RA *et al*. for more details.^20^

### Instrumental variable estimators

In our primary analyses, we report the estimates of the effect of educational attainment using two-stage least squares for continuous outcomes and additive structural mean models for binary outcomes.^21–23^ These identify the causal mean and risk differences, respectively. The primary analyses adjust for sex, age and month of birth. In the Supplementary Materials we report sensitivity analyses without adjustment, and adjusting for the covariates associated with the educational attainment genetic score.

#### Identifying assumptions

The two-stage least squares estimates of the effect on the continuous outcomes can be point identified by assuming a constant effect of education on the outcome, i.e. that an additional year of education causes the same unit change in the outcome for everyone. Alternatively, we could assume that the education allele score has a monotonic effect on education. That is, an additional educational attainment-associated allele will increase the likelihood of having a higher level of education in everyone. The estimate then identifies the ‘local average treatment effect’ (LATE). This parameter is the effect of education on individuals whose educational attainment was affected by the score. For the structural mean models for binary outcomes, we can either assume monotonicity, interpreted in the same way as above, or that the effects of each year of education are the same irrespective of how many education variants each participant has.^24^ These estimates can be interpreted as the effect of education on individuals who chose to receive a given level of education.

#### Sensitivity analyses

We conducted analyses to investigate the sensitivity of our results to weighting for non-random sampling, the reduced form, robustness to adjustment, assumption about the structure of pleiotropy, and within-family estimators; see Supplementary Material for details.

## Results

### Descriptive statistics

The UK Biobank invited 9.2 million people aged between 40–69 to attend 23 centres across Great Britain (Island of England, Wales and Scotland).^25^ Of those invited, 503 317 (5.47%) were recruited and gave consent over 2006–10 for the study. Of these, 315 436 met the inclusion criteria for this study. See the Supplementary Material for a flowchart of the inclusions and exclusion of participants and Supplementary Figure S2, available as Supplementary data at *IJE* online. The average age when attending the assessment centre was 56.9, and 53.8% were female. On average, UK Biobank participants were more educated than the British population; 41.0%, 64.0% and 82.1% had a degree or equivalent, had post-16 education and had any academic qualifications, respectively. The UK census found that 27.9%, 61.8% and 76.5% of the British population aged between 40 and 70 in 2011 had these qualifications, respectively.^26^ See Table 1 for a description of the participants included in this study. We used inverse probability weights to correct this selection.

**Table 1.** Characteristics of 315 436 participants of the UK Biobank

Characteristic	Number	Proportion	Count
Male	315 436	0.46	146 571
**Characteristic**	**Number**	**Mean**	**SD**
Year of birth	315 436	1951	8
Age left education (eduyears)	315 436	18.19	3
**Outcome**	**Number**	**Proportion**	**Count**
Hypertension	307 496	0.25	76 638
Diabetes	313 766	0.04	13 877
Stroke	314 978	0.02	4 772
Heart attack	314 978	0.02	7 175
Depression	300 594	0.15	44 283
Cancer	314 152	0.13	40 014
Died	315 436	0.02	5 340
Ever smoked	314 422	0.10	31 259
Currently smoke	314 422	0.45	141 825
Income over £18k	274 617	0.78	215 423
Income over £31k	274 617	0.53	145 685
Income over £52k	274 617	0.27	72 867
Income over £100k	274 617	0.06	15 190
**Outcome**	**Number**	**Mean**	**SD**
Grip strength (kg)	314 788	0.33	10.89
Arterial stiffness	113 856	0.02	4.07
Height (cm)	314 760	168.92	9.26
BMI (kg/m^2^)	314 455	27.36	4.75
Diastolic blood pressure (mmHg)	297 872	82.25	10.12
Systolic blood pressure (mmHg)	297 871	138.11	18.62
Intelligence (0 to 13)	113 033	6.25	2.10
Happiness (0 to 5 Likert)	114 971	3.45	0.70
Alcohol consumption (0 low to 5 high)	315 239	3.16	1.48
Television watching (h/day)	304 230	2.86	1.63
Moderate exercise (days/week)	301 195	3.61	2.33
Vigorous exercise (days/week)	301 440	1.82	1.94

BMI, body mass index; SD, standard deviation.

### Testing the assumptions

**Figure 1.**
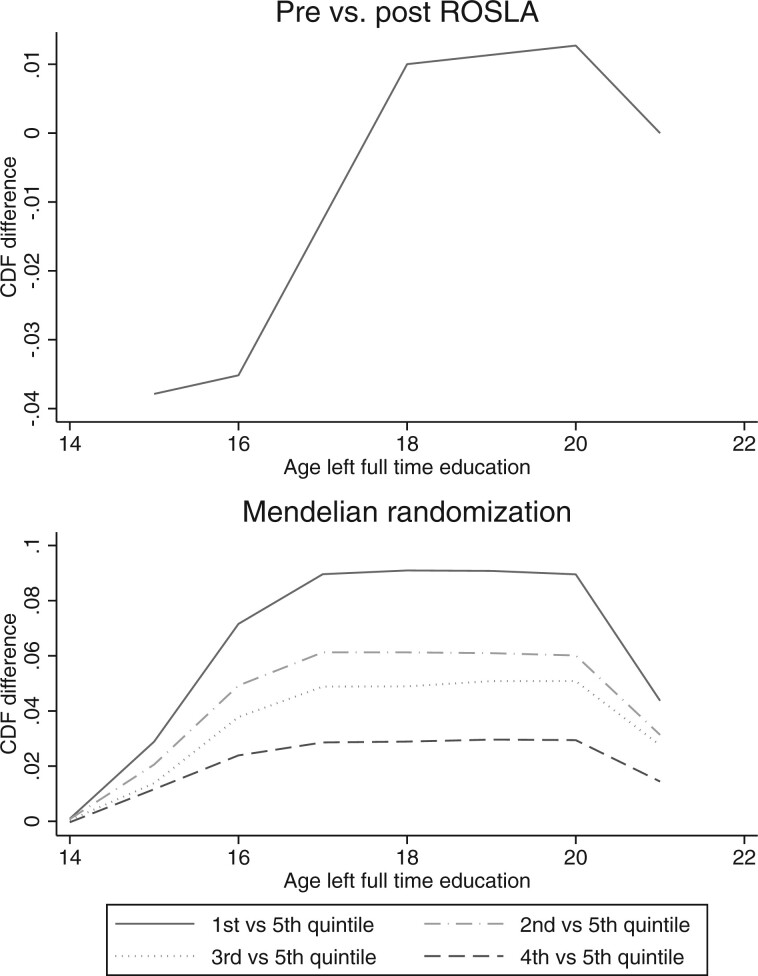
Differences in the age participants left school across the raising of the school leaving age (top) and quintiles of the educational attainment genetic score (bottom). The policy reform substantially reduced the proportion who left school before age 16. The genetic scores are associated with a higher probability of remaining in schools at all ages. ROSLA, Raising Of School Leaving Age

Participants born after August 1957, who were affected by the raising of the school leaving age, were 23.0 [95% confidence interval (95% CI): 21.7 to 24.4] percentage points more likely to remain in school after age 15 than those born before September 1957, who were not affected by the reform. We used the 74 genetic variants detected in the educational attainment GWAS to construct a weighted genetic score in the UK Biobank. Each variant was weighted by its association with educational attainment in the discovery sample of the GWAS. The educational attainment allele score was more weakly associated with educational attainment than the policy reform. A unit increase in the score was associated with 1.45 additional years of education (95% CI: 1.36 to 1.55) as defined by the International Standard Classification of Education (ISCED). Thus, the educational attainment allele score was a strong instrument (max partial F statistic = 1099) but explained less of the variation in educational attainment than the raising of the school leaving age (max partial F statistic = 2182). Neither proposed instrument is likely to suffer from weak instrument bias. The policy reform induced fewer individuals to leave school before the age of 16 (Figure 1, top). The educational attainment allele score was associated with an increased likelihood of remaining in school at all ages (Figure 1, bottom). We assessed the relative bias of the estimators using covariate balance plot; see Supplementary Material for details.

### Effect of educational attainment on outcomes

**Figure 2.**
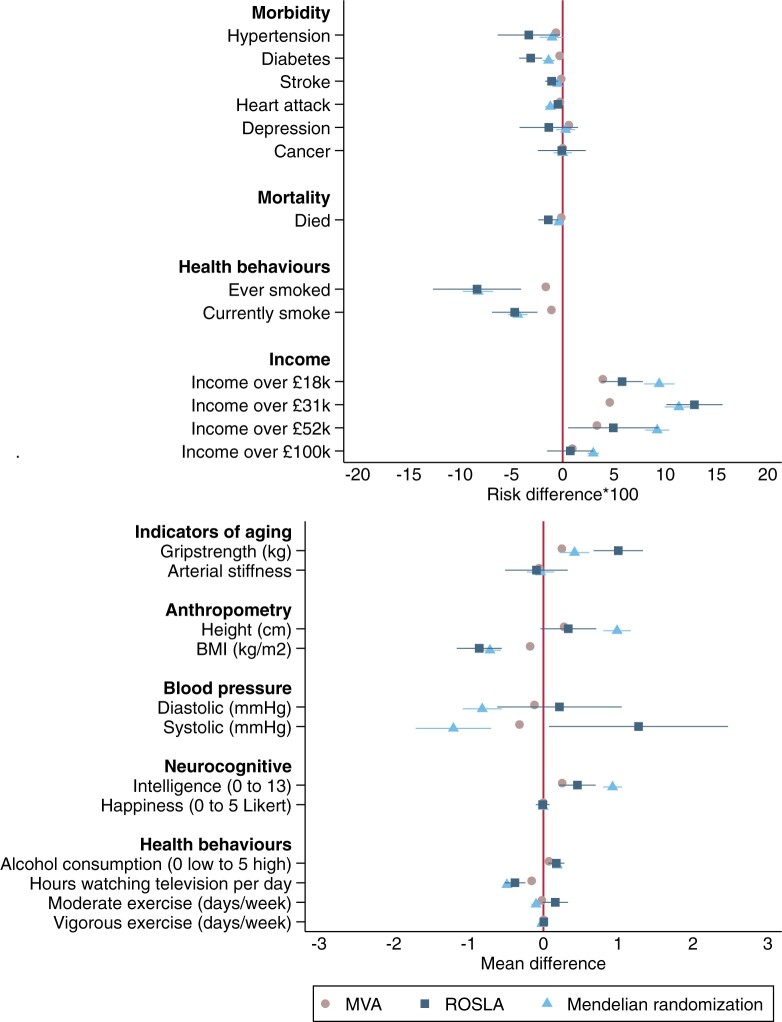
The effect of one additional year of schooling on morbidity, mortality and socioeconomic outcomes, estimated via multivariable-adjusted regression (MVA), using instrumental variables raising of the school leaving age, and the Mendelian randomization and educational attainment genetic score. The results were similar using the educational allele score and raising the school leaving age. They suggested that the multivariable adjusted difference is likely to underestimate the difference in outcomes caused by education. Adjusted for month and year of birth, sex and the 10 principal components of population stratification. Confidence intervals allow for clustering by month of birth and sample weighted to adjust for under-sampling of less educated. ROSLA estimates are the effects of an additional school year rather than the reform's effect. ROSLA, Raising Of School Leaving Age; ISCED, International Standard Classification of Education

Figure 2 plots the estimated effects of an additional year of education on each of the 25 outcomes.

#### Mortality

Each additional year of education was observationally associated with -0.14 (95% CI: -0.16 to -0.11) percentage points lower mortality. The Mendelian randomization estimates were similar but less precise at -0.37 (95% CI: -0.80 to 0.06). This effect is larger than the observational association of educational attainment and mortality but smaller than the effect of remaining in school estimated by the raising of the school leaving age at -1.40 (95% CI: -2.38 to -0.43) (Figure 2).

#### Morbidity

Observationally, an additional year of education was generally associated with improved health. Each year of education was associated with 0.65 per 100 (95% CI: 0.58 to 0.72) fewer cases of high blood pressure, 0.30 (95% CI: 0.27 to 0.34) fewer diagnoses of diabetes, 0.14 (0.12 to 0.17) fewer strokes, 0.27 (95% CI: 0.24 to 0.30) fewer heart attacks and 0.60 (95% CI: 0.55 to 0.66) more episodes of depression. There was little evidence of differences in rates of cancer diagnoses. The Mendelian randomization estimates suggested that each year of education reduced the likelihood of being diagnosed with high blood pressure by 1.04 per 100 (95% CI: -0.18 to 2.25), diabetes by 1.38 (95% CI: 0.78 to 1.97), stroke by 0.50 (95% CI: 0.14 to 0.86) and heart attack by 1.21 (95% CI: 0.70 to 1.71). However, the Mendelian randomization estimates provided little evidence of an effect on depression or cancer. The policy reform estimates were in the same direction as the Mendelian randomization results and provided little evidence of effects on depression or cancer, but implied smaller effects on heart attacks.

#### Health behaviours

An additional year of education was associated with 1.65 per 100 (95% CI: 1.57 to 1.73) and 1.10 (95% CI: 1.04 to 1.17) fewer ever and current smokers. The Mendelian randomization analysis suggested that the effects were substantially larger at 8.25 (95% CI: 6.78 to 9.73) and 4.38 (95% CI: 3.43 to 5.34) fewer ever and current smokers per 100, respectively. The estimates based on the raising of the school leaving age were similar to those using Mendelian randomization. Each year of education was associated with a 0.07 (95% CI: 0.07 to 0.08) unit increase in alcohol consumption. The Mendelian randomization estimates implied that the causal effect of an additional year of schooling was 0.19 (95% CI: 0.15 to 0.23). Each year of education was associated with watching 0.16 (95% CI: 0.15 to 0.16) fewer hours of television per day. The Mendelian randomization suggests that this likely underestimates the causal effects at 0.49 (95% CI: 0.44 to 0.54). A year of education was associated with 0.02 (95% CI: 0.02 to 0.02) fewer days per week of moderate exercise. The Mendelian randomization estimate suggested this underestimated the causal effect at 0.10 (95% CI: 0.04 to 0.16). There were only very small associations between educational attainment and vigorous exercise, similar to the Mendelian randomization and policy reform estimates.

#### Income

Each additional year of education was associated with a higher risk of having an income above £18 000, £31 000, £52 000 and £100 000 of 3.91 (95% CI: 3.74 to 4.06), 4.59 (95% CI: 4.51 to 4.67), 3.34 (95% CI: 3.20 to 3.48) and 0.94 (95% CI: 0.88 to 1.00) per 100 participants, respectively, on the absolute risk difference×100 scale. The Mendelian randomization estimates were larger, suggesting 9.42 (95% CI: 7.93 to 10.90), 11.33 (95% CI: 9.94 to 12.72), 9.22 (95% CI: 8.06 to 10.38) and 2.98 (95% CI: 2.44 to 3.53) increase per 100 participants, respectively. The raising of the school leaving age estimates were similar in direction and magnitude to the Mendelian randomization estimates but provided little evidence that education affected the probability of having the highest income.

#### Indicators of ageing

Each year of education was associated with an average 0.25 kg (95% CI: 0.24 to 0.26) stronger grip. The Mendelian randomization estimates suggest a larger causal effect of 0.42 kg (95% CI: 0.22 to 0.61). Education was also associated with 0.06 (95% CI: 0.04 to 0.07) lower arterial stiffness. The Mendelian randomization estimate was imprecise but in the same direction, implying that each year of education reduced arterial stiffness by 0.04 (95% CI: -0.14 to 0.22). The estimates based on the raising of the school leaving age suggested a larger effect on grip strength but similar equivocal effects on arterial stiffness.

#### Anthropometry

Each additional year of education was observationally associated with a 0.28 (95% CI: 0.27 to 0.29)-cm increase in height and 0.18 (95% CI: 0.17 to 0.18)-kg/m^2^ reduction in BMI. The Mendelian randomization estimates suggested larger causal effects of education of 0.99 (95% CI: 0.80 to 1.17)-cm increase in height and a 0.71 (95% CI: 0.57 to 0.86)-kg/m^2^ reduction in BMI. The estimated effect on height using the raising of the school leaving age was very similar to the observational association. The effects on BMI estimated using the reform were much larger than the observational associations, and very similar to the Mendelian randomization estimates. The effect of education on height is likely due to pleiotropic or residual population stratification. We investigated this using a negative control outcome: whether the participant reported being taller than average at age 10. Mendelian randomization implied that each additional year of education was associated with being 4.14 (95% CI: 3.03 to 5.24) percentage points more likely to report being taller than average at age 10. We investigated this finding further in the pleiotropy robust sensitivity analyses below.

#### Blood pressure

Each additional year of education was associated with lower diastolic and systolic blood pressure (0.12 mmHg, 95% CI: 0.10 to 0.14 and 0.32 mmHg, 95% CI: 0.29 to 0.35, respectively). The genetic analysis suggested the causal effects were in the same direction but larger (0.82 mmHg, 95% CI: 0.56 to 1.08 and 1.20 mmHg, 95% CI: 0.70 to 1.71, respectively). There was little evidence that the reform affected diastolic blood pressure and some evidence that it increased systolic blood pressure. However, these estimates are likely to be due to age effects, as they were consistent with the average year-on-year differences in systolic blood pressure.^13^

#### Neurocognitive

Each year of education was associated with 0.25 (95% CI: 0.25 to 0.26) additional correct answers on the intelligence test, but there was little difference in subjective wellbeing. The Mendelian randomization estimates suggested that educational attainment caused 0.93 (95% CI: 0.80 to 1.05) additional correct answers but found little detectable effect on subjective wellbeing. The estimates of the effect on intelligence based on the raising of the school leaving age was also positive but were slightly smaller. There was little evidence that the reform affected subjective wellbeing.

### Sensitivity analyses

See Supplementary Material for sensitivity analyses. The results were largely robust to weighting, the reduced form, adjustment for covariates, a range of pleiotropy robust methods and within-family fixed effects, but there was some evidence of effect heterogeneity.

## Discussion

Our findings suggest that the differences in many later life outcomes between educational groups are likely caused by education. Our Mendelian randomization estimates suggest educational attainment affects morbidity, including reducing the risk of hypertension, diabetes, stroke, heart attack and mortality. Furthermore, these results imply that education reduces the risk of currently or ever smoking, increases household income, lowers blood pressure and increases scores on intelligence tests.^27^ However, there was evidence that education reduced rates of moderate exercise and increased alcohol consumption. Our sensitivity analyses suggest that confounding by genotypic or phenotypic confounders or horizontal pleiotropy are unlikely to explain our results.

Triangulating across multiple sources of evidence can help provide stronger evidence of causal effects.^28^ Here, we found that the two natural experiments gave similar results. The two sources of variation, Mendelian randomization and the raising of the school leaving age, have distinct causes of and directions of bias. The similarity in results strengthens the case that education has causal effects. The raising of the school leaving age affected relatively low-ability students, who were forced to remain in school for an additional year.^1^ In contrast, our Mendelian randomization results exploit variation across the entire distribution of educational attainment, estimating an average effect of an additional year of schooling for everyone from those who leave school at 15 to graduates (see Figure 1).^29^ A priori, there was little reason to assume that a year of additional schooling would have the same effects on a high school leaver as on a graduate. Surprisingly we found relatively little evidence that the effects of educational attainment differed across the different estimates. The estimates from the two natural experiments are similar, both in direction and, in many cases, magnitude. There was very little evidence of heterogeneity in the effects identified by different variants. The effect of an additional year of education on smoking is comparable to other studies using natural experiments. For example, Grimard and Parent (2007) used data from the US Current Population Survey and the Vietnam draft to estimate that in 1995–99 an additional year of schooling caused a 7.97 (95% CI: 3.15 to 12.79) and 11.13 (95% CI: 5.54 to 16.72) percentage point reduction in probability of currently or ever smoking, respectively.^30^ The results were very different for other outcomes, such as measured blood pressure. The effects of education on blood pressure estimated using the raising of the school leaving age may reflect non-linear cohort effects as previously discussed.^13^

We found evidence that the educational attainment polygenic score correlated with baseline covariates, including birthweight, being taller than average at age 10, whether the mother smoked during pregnancy, parental mortality and geography. These associations may reflect dynastic effects or assortative mating (Supplementary Figure S4, available as Supplementary data at *IJE* online). If there is assortative mating, then this could induce associations between education variants and variants for other traits. For example, if highly educated people assortatively mate with taller spouses, then the Mendelian randomization estimates of the effect of education on height would be positively biased. These effects may explain the implausible Mendelian randomization estimate of the effect of education on height. A sub-sample (*n* = 310 230) of the study provided information on whether they were taller than average at age 10; temporally, this variable cannot be affected by completed years of educational attainment. When we adjusted for being taller than average at age 10, the estimated effect falls from 0.93 (95% CI: 0.78 to 1.09) to 0.62 (95% CI: 0.46 to 0.78) cm increase in height per year of education. This result suggests that assortative mating, dynastic effects or population stratification may explain the estimated effects of education on height. We investigated whether we could use the siblings in the UK Biobank and family fixed effects estimators to account for these sources of bias (Supplementary Figure S8, available as Supplementary data at *IJE* online). However, the power of this analysis needed to be higher to draw meaningful conclusions. Brumpton and colleagues found little evidence that height and BMI affected educational attainment when using a within-family design and a larger sample of siblings.^31^ Our sensitivity analyses suggested that pleiotropy was unlikely to explain our results (see Supplementary Material).

We used a single study and individual participant data, which allowed us to compare different methods for estimating the effects of educational attainment in the same study. However, the sample size and number of events for some disease outcomes were relatively small. This limits our power to detect effects relative to two-sample Mendelian randomization studies, which can use data from many more cases. This may explain why we could not detect an effect of educational attainment on overall cancer rates. This is surprising, given the differences in smoking rates we report and other two-sample Mendelian randomization studies which have reported protective effects of educational attainment on lung cancer.^32^ However, we were not adequately powered to detect effects on cancer subtypes.

## Conclusion

In conclusion, two independent natural experiments suggest that education has wide-ranging effects on important outcomes measured much later in life. Importantly, the two experiments affected educational outcomes differently—one exclusively affected those at the bottom of the distribution, the other affected education levels across the whole distribution—and yet found effects of a similar magnitude. This suggests a common treatment effect of additional education on many health behaviours and outcomes.

## Ethics approval

UK Biobank has ethical approval from the North West Multi-centre Research Ethics Committee. At the touchscreen, all participants gave informed consent using a signature-capture device. This research was conducted using the UK Biobank Resource using application 8786.

## Supplementary Material

dyad104_Supplementary_Data

## Data Availability

The data used in the study are available from the UK Biobank study. Please get in touch with [access@ukbiobank.ac.uk] for further information. All analyses were conducted in StataMP 14.0.^33^ The code used to generate these results has been archived at [https://github.com/nmdavies/UKbiobank-MR-vs-ROSLA].
