# Supplementary material for: The causal effects of education on adult health, mortality and income: evidence from Mendelian randomization and the raising of the school leaving age
Source: Int J Epidemiol. 2023 Jul 18;52(6):1878–86. doi: 10.1093/ije/dyad104 (PMC10749779; doi:10.1093/ije/dyad104)
Supplement: dyad104_Supplementary_Data [file dyad104_supplementary_data.docx]

# Supplementary Materials

# Review of Mendelian randomization estimating the effects of education

Legal changes to school leaving ages and genetics have been used as instrumental variables for education. However, few studies have systematically compared the estimates from policy reforms and Mendelian randomization.^1^ The instrumental variable assumptions are plausible for Mendelian randomization analysis of phenotypes whose biological pathways are relatively well understood (e.g. variants in the CRP gene for CRP levels^2^ or variants in ALDH2 for alcohol^3^), they may be less plausible for phenotypes where the mediating pathways are less well understood such as education. For example, genetic variants that affect parents’ education may have direct effects on the offspring (so-called “dynastic effects”); assortative mating by education^4^; or that more educated parents have different ancestry from those with less education. These potential sources of bias are illustrated in **Supplementary Figure S1**. While Mendelian randomization using samples of unrelated individuals may be a credible identification strategy for biologically proximal phenotypes such as CRP or alcohol consumption, it may be less plausible for biologically distal phenotypes such as education. Recent studies have used genetic variants associated with education to estimate the effects of education on coronary heart disease and dementia.^5,6^ However, unlike hypotheses that relate to biological traits, such as lipids, there are no randomized trials that can provide gold-standard evidence of the causal effects of education. Mendelian randomization has been widely used in the literature to estimate the effect of educational attainment. **Supplementary Table S1** summarises 81 papers published up to January 2023 which include “Mendelian randomization” and “educational attainment” on pubmed, excluding methods and review papers. Despite this volume of research papers, no other papers have directly compared estimates from the raising of the school leaving age and Mendelian randomization. Here, we address this gap in the literature.

Two key questions in the scientific and policy literature are whether the effects of education across individuals or at different points in the life course are heterogeneous.^7–10^ For example, does an additional year of schooling at age 16 have the same effect on everyone? Does an additional year of schooling at age 16 have the same effect as an additional year of schooling at age 20? Many of the previously investigated policy reforms affect a subset of individuals at a specific age (e.g. the effect of an additional year of education for low-ability students at age 16). Policymakers may be interested in the effects of education on average across the whole population or of the effects of obtaining a specific length of schooling (e.g. staying in school to age 18 versus 16). However, genetic variants affect educational attainment across the entire lifespan. They identify the average effect of an additional year of school across the entire cohort. Furthermore, while many previous studies^11^ have used multivariable adjustment, policy reforms^12^, or Mendelian randomization to estimate the effects of educational attainment on outcomes later in life, previous studies have not directly compared estimates from policy reforms and genetic instrumental variables (see **Supplementary Table S1** for a summary of papers).

# Data

## Genotyping

DNA was extracted from the blood samples which were genotyped using UK BiLEVE Axiom and UK Biobank Axiom arrays. Full details of the genotyping and imputation procedure are available.^13^ The genotyping data quality control process consisted of the following steps. First, SNPs were set to missing when there was evidence of clustering across chips, batch or plate effects, a departure from Hardy Weinberg equilibrium, sex effects, array effects or discordance across the genotyping controls (at p<10^-12^). On average, these exclusion criteria affected 7,704 (0.01%) of SNPs per batch. Second, 968 participants with less than 5% call rates or extreme heterozygosity were excluded. Third, individuals with European ancestry were identified by projecting each participant onto the principal components from the 1000 genomes project. Fourth, we excluded participants who were more than third-degree related. Fifth, we excluded participants whose self-reported gender did not match their genetic sex.

# Bias assessment

## Methods

Covariate balance plots allow for direct comparison of the relative bias caused by omitting each observed covariate from an instrumental variable or multivariable-adjusted regression analysis. The third instrumental variable assumption, exclusion, that the instrumental variable only affects the outcome via the exposure interest, cannot be directly tested. This is because we cannot know whether we have measured or tested all potential pathways. However, this assumption can potentially be investigated (falsified) if there are measures of alternative mediating pathways. The UK Biobank recruited participants over the age of 40 after they completed full-time education; therefore, it has few suitable measures to evaluate this assumption. We evaluated this assumption using a small number of available covariates and a set of genetic covariates.

Previous studies have found that the raising of the school leaving age affected students who would otherwise have left school at the age of 15. These students were generally less academic, and most chose to leave at the new minimum age of 16. This meant the reform had little effect on the proportion of students remaining in school to 18 or attending university. This means the raising of the school leaving age estimates the effect of an additional year of schooling at age 15. In contrast, the genetic variants associated with years of education are likely to associate with educational outcomes across the entire life course, the probability of remaining in school at age 15, 16, 18 and the likelihood of obtaining a degree. The effect identified by the genetic variants is therefore a weighted average of the effects of an additional year of educational attainment across the entire education distribution.^14,15^ We investigated this by plotting the difference in the cumulative density of educational attainment by the policy reform and between the quintiles of the educational attainment allele score.

### Covariates: Non-genetic

We also assessed the association of the proposed instruments with 14 phenotypes that are measures of circumstances around the time of birth, childhood, or relating to family background. We assessed: birth location (easting, northing, Index of Multiple Deprivation, urban vs rural and distance from London), early life factors (birth weight, whether the participant was breastfed, or their mother smoked during pregnancy, their comparative body size and height at age 10, and their number of brothers and sisters). Some of these variables occurred after conception. However, they are unlikely to be directly affected by the educational attainment of the participant, so they can provide helpful evidence of the plausibility of the instrumental variable assumptions.

### Covariates: Genetic

Genomes are determined at conception. Mendel’s laws of *independent assortment* and *segregation* state that in the absence of any other process (e.g. assortative mating, sample selection bias, variants in linkage disequilibrium, or pleiotropy) genetic variants for one trait will be inherited independently of another. Thus people who have many or few education increasing alleles should, on average, have a similar number of alleles known to increase BMI. However, individual SNPs are unlikely to provide sufficient statistical power. One way to increase the power is to assess this assumption using multiple variants for a given trait combined with polygenic risk scores. For example, if Mendel’s law of independent assortment holds, we would expect a polygenic risk score for educational attainment to be independent of a polygenic risk score for BMI. If the education polygenic score associates with scores for other traits, then it suggests that Mendel’s laws are unlikely to hold or the sample is affected by selection bias.

We evaluated this using genetic scores for 45 traits extracted from MR-Base (**Supplementary Table S2**).^16^ We constructed the scores from extracted SNPs associated with each trait at p<5×10^-5^. We used a lower threshold than is usually used for genome-wide significance (p<5×10^-8^) to define the scores because we wanted to maximise the explanatory power of the scores. Furthermore, the educational attainment genetic score cannot have pleiotropic effects on the other polygenic scores. We LD pruned the SNPs for each trait using a threshold of r^2^>0.001 across a distance of 10,000kb. We excluded SNPs from these scores that were correlated with (i.e. in linkage disequilibrium with) the 74 SNPs we used as instrumental variables for educational attainment (LD >r^2^>0.001).^17^ This resulted in a set of SNPs at independent points in the genome for each trait. We constructed allele scores equal to the sum of the effect alleles for each trait.^18^ The contribution of each SNP to the allele score was weighted by the coefficient reported in the GWAS for that trait. We harmonised the direction of SNP effects between the UK Biobank and the GWAS. Finally, we checked for consistency of the allele frequency reported in the GWAS and the UK Biobank data.

## Covariate balance plots

We were concerned that our results may be affected by selection bias or residual confounding. If there was a strong selection into the study, this could induce correlations between the instruments and outcomes that are independent in the population. We evaluated this using covariate balance plots.^19^ Covariate balance plots compare the relative bias of the instrumental variable and conventional estimators if an observed covariate was omitted. We assessed the bias associated with 14 non-genetic phenotypes and polygenic scores for 45 traits. The biases for the educational attainment genetic score were similar in size to those for the raising of the school leaving age (**Supplementary Figures 3** and **4**).

### Phenotypic confounders

The parents of participants affected by the raising of the school leaving age were less likely to have died. These differences are likely to be due to cohort effects. On average, participants affected by the reform were one year younger than those who were not affected. Offspring educational attainment may also affect parental mortality.^20–22^ There was little evidence that the reform affected any other baseline and childhood phenotypes. The educational attainment genetic score was non-randomly distributed across the UK (**Supplementary** **Figure S4**). On average, genetic variants associated with educational attainment were more common in the east and south of the UK. However, the magnitude of these associations was relatively small. There was evidence that the educational attainment genetic score was associated with having been breastfed, birthweight, being taller than average at age 10, and whether the participants’ mother smoked in pregnancy. These associations may be driven by dynastic effects or assortative mating. Dynastic effects could occur because, on average, participants with more education-associated genetic variants will have more educated parents. If more educated parents behave differently, e.g. smoke less in pregnancy, then this could cause an association with the educational attainment genetic score. Assortative mating could induce associations if, on average, more educated parents choose taller spouses. Nevertheless, these covariates are only weakly associated with the outcomes. There was little evidence that the covariates associate more strongly with the educational attainment genetic score than the reform. As a result, for many outcomes, the bias from these covariates is small; for more details, see the full adjusted sensitivity analyses below. This suggests that residual confounding due to phenotypic covariates is unlikely.

### Genetic confounders

The educational attainment genetic score weakly associated with polygenic scores for other phenotypes, including bipolar disorder, childhood intelligence, inspection time, simple reaction time and infant head circumference (**Supplementary** **Figure S4**). However, there was little evidence that bias components for the educational attainment genetic score were larger than those for the raising of the school leaving age. These estimates suggest that genotypic confounding is unlikely to be a substantial source of bias.

## Covariate balance plots

We were concerned that our results may be affected by selection bias or residual confounding. If there was a strong selection into the study, this could induce correlations between the instruments and outcomes that are independent in the population. We evaluated this using covariate balance plots.^19^ Covariate balance plots compare the relative bias of the instrumental variable and conventional estimators if an observed covariate was omitted. We assessed the bias associated with 14 non-genetic phenotypes and polygenic scores for 45 traits. The biases for the educational attainment genetic score were similar in size to those for the raising of the school leaving age (**Figures 2** and **3**).

### Phenotypic confounders

The parents of participants affected by the raising of the school leaving age were less likely to have died. These differences are likely to be due to cohort effects. On average, participants affected by the reform were one year younger than those who were not affected. Offspring educational attainment may also affect parental mortality.^20–22^ There was little evidence that the reform affected any other baseline and childhood phenotypes. The educational attainment genetic score was non-randomly distributed across the UK (**Supplementary** **Figure S3**). On average, genetic variants associated with educational attainment were more common in the east and south of the UK. However, the magnitude of these associations was relatively small. There was evidence that the educational attainment genetic score was associated with having been breastfed, birthweight, being taller than average at age 10, and whether the participants’ mother smoked in pregnancy. These associations may be driven by dynastic effects or assortative mating. Dynastic effects could occur because, on average, participants with more education-associated genetic variants will have more educated parents. If more educated parents behave differently, e.g. smoke less in pregnancy, then this could cause an association with the educational attainment genetic score. Assortative mating could induce associations if, on average, more educated parents choose taller spouses. Nevertheless, these covariates are only weakly associated with the outcomes. There was little evidence that the covariates associate more strongly with the educational attainment genetic score than the reform. As a result, for many outcomes, the bias from these covariates is small; for more details, see the full adjusted sensitivity analyses below. This suggests that residual confounding due to phenotypic covariates is unlikely.

### Genetic confounders

The educational attainment genetic score weakly associated with polygenic scores for other phenotypes, including bipolar disorder, childhood intelligence, inspection time, simple reaction time and infant head circumference (**Supplementary** **Figure S4**). However, there was little evidence that bias components for the educational attainment genetic score were larger than those for the raising of the school leaving age. These estimates suggest that genotypic confounding is unlikely to be a substantial source of bias.

# Sensitivity analyses

## Methods

We investigated the raw association of the education score and each outcome, the so-called “reduced form”. The reduced form is a general test of causation under a minimal set of assumptions, i.e. it does not require point identification. Reduced form and instrumental variable estimates should be consistent in direction. We were concerned that our results could suffer from residual confounding given the non-random geographic distribution of alleles. We therefore investigated whether our results were sensitive to removing the basic controls (sex, year and month of birth, the first ten genetic principal components). We also investigated whether our results were sensitive to including a richer set of controls in the subset of participants with observations on these.

Our results could be affected by bias due to the pleiotropic effects of the variants. That is, if the variants directly affected the outcomes via pathways other than education. We investigated this using MR-Egger, weighted median, and weighted mode estimators in summary data analyses.^23–25^ We included the inverse variance weighted estimates for comparison. These estimates use the educational attainment GWAS discovery sample coefficients. We assessed the variability in the estimated effect of education across the 74 genetic variants using the I^2^ statistic.^26^

We investigated the robustness of our results to assortative mating, dynastic effects and residual population stratification by including a family fixed effect and clustering the standard errors by family. This analysis is restricted to the sample of UK Biobank participants who had siblings in the study. For each outcome we restricted this analysis to participants with siblings with non-missing values of the outcome. A minority of the sample had siblings. Hence the power of this analysis is much smaller than in the primary analysis above. We used the Stata command xtivreg. This estimator does not allow weights. Therefore we could not adjust for sampling in this analysis. However, weighting made little material difference to the Mendelian randomization estimates using unrelated individuals. Therefore, inverse probability weights are unlikely to affect this analysis.

## Weighting to account for non-random sampling

Reanalysing the data without applying inverse probability weights did not materially influence the Mendelian randomization estimates, see **Supplementary Figure S5**.

## Association between the educational attainment genetic score and the outcome, the “reduced form”

The associations between the educational attainment genetic score and the 25 outcomes are shown in **Supplementary Figure S6**. These associations are consistent with the main instrumental variable results presented above.

## Robustness of results to adjustment

We investigated whether the results were affected by removing the covariates, including sex, year and month of birth, and the first ten principal components of population stratification. The only estimates affected by this were grip strength and height, which attenuated towards the null. See **Supplementary Figure S7** for details. There was little detectable impact of adjusting for a range of confounders; see **Supplementary Figure S8**. The estimated effect of height attenuated modestly. These sensitivity analyses suggest that residual confounding is unlikely to explain our results.

## Pleiotropy robust methods

We investigated whether the results could be explained by pleiotropy using MR-Egger, weighted median and weighted mode approaches (**Supplementary Figure S9**). MR-Egger was highly imprecise for all outcomes and provided few inferences. The different estimators provided consistent evidence of causal effects for some outcomes, including diabetes, heart attack, mortality, smoking, income, grip strength, BMI, blood pressure, intelligence, alcohol consumption, and exercise. There was evidence of differences in the estimates for height, which may indicate that the inverse variance weighted and two-stage least squares estimates above suffer from pleiotropy. The weighted mode estimator suggested little effect of educational attainment on height. We present the I^2^ statistics of the heterogeneity in the estimated effects of education across the 74 genetic variants in **Supplementary Table S3**.

## Sibling fixed effects models

We investigated whether our results were robust to including family fixed effects in the sub-sample of participants with siblings. Many of these siblings were excluded from the primary analysis above. Family fixed effects account for all between family differences in outcomes. This analysis is robust to bias from assortative mating, dynastic effects and residual population stratification. However, the power of the analysis is far lower because it only uses a tenth of the sample and half of the genetic variation that occurs within families. Hence these results were very imprecise. We found little evidence that these within-families estimates differed from the estimates from unrelated individuals presented above and little evidence that they differed from the null (see **Supplementary Figure S10**). The unadjusted association of educational attainment and the outcomes can be found in **Supplementary Table S4**.

# Discussion of sensitivity analyses

## Pleiotropy

A further limitation is that the genetic variants may have pleiotropic or direct effects on the outcomes, as their biological mechanisms of effect are unknown. However, our estimates were similar when using the weighted median and mode estimates. An exception to this was height, where the mode and median-based estimate suggested smaller effects. A further limitation is that we report binary outcomes for mortality, future studies could use instrumental variable estimators for survival outcomes to increase statistical power.^27^

## Selection bias, assortative mating and dynastic effects

A limitation of our study is that we used a non-representative sample. We have addressed this using inverse probability weights. The weights made little difference to the Mendelian randomization estimates, suggesting that sample selection bias on educational attainment is unlikely to affect our results. However, strong selection on the polygenic index for educational attainment, which was independent of selection on educational attainment could still affect our estimates. The Mendelian randomization estimates can suffer from bias due to assortative mating or dynastic effects; however, except for height, adjusting for measured baseline covariates had little effect on our results (**Supplementary Figures S5** and **6**). Our results could reflect either direct effects of the participants’ educational attainment, assortative mating between their parents, dynastic effects of their parents’ education or differences in ancestry not accounted for by the principal components (**Supplementary Figure S1**). These potential explanations could be evaluated using either offspring-mother-father trios or sibling designs. Okbay and colleagues (2016) found little evidence that the effects of the genome-wide significant education variants attenuated after controlling for family structure.^17^ However, these analyses may not have had sufficient power to detect dynastic effects. Kong and colleagues investigated this using a sample of parents and offspring from Iceland.^28^ They found that a polygenic score for education, made up of alleles that were not inherited, was associated with offspring’s education. The association with the non-inherited polygenic score was 29% of the size of the association with the inherited genetic score. This suggests that the effects we identify are likely to represent a combination of the effect of the participants’ education and their parents’ education. Kong and colleagues’ results provide an upper bound for the contribution of parents’ education of 29%. The contribution of parents’ education to our results will be smaller if the direct effect of parents’ education on each outcome is smaller than the effect of the participant’s education. Howe and colleagues found that the genetic association with educational attainment were (47%; 41%, 52%) attenuated using within-family estimators and a large sample of siblings. Some Mendelian randomization estimates, such as the effects of height and BMI on education are biased using results from samples of unrelated individuals, other estimates were largely unbiased, for example, the effects of BMI, cigarette smoking, and systolic blood pressure and on all-cause mortality.^29,30^ Determining the relative contributions of parent versus offspring education will require large samples of parent-offspring data.^31^

Supplementary Figure S1: The Mendelian randomization estimates identify the effects of education on the outcomes using directly inherited genetic variants known to associate with educational attainment. These effects could be mediated via 1) an effect of the participants’ educational attainment on their later outcomes, 2) a direct effect of parents’ phenotypes on their offspring’s outcomes, 3) or assortative mating on education and other phenotypes (e.g. height) between parents. Dynastic effects would lead to the Mendelian randomization estimates to overestimate the direct effects of educational attainment. Assortative mating would induce associations between the 74 SNP known to associate with education and other variants associated with education or related traits across the genome.

Assortative

mating

Dynastic effect

Outcomes

Dynastic effect

Participant’s

educational

attainment

Inherited genetic variants

Mother’s

educational

attainment

Father’s

educational

attainment

### Supplementary Figure S2: Selection of participants into the study

19,538 without measures of education

315,436 with measures of educational attainment

334,974 with consent

334,980 Unrelated

408,258 Europeans

486,566 included

487,409 had genotyping data

15,255 had no genotype data

participants consented

8,735,136 did not respond or consent

participants consented

653 did not provide data or withdrew

participants consented

502,664 provided data

6 withdrew consent

73,276 related

78,308 non-European

843 had sex mismatches, sex chromosome aneuploidy, or excess heterozygosity

503,317 consented

9,238,453 invited

Supplementary Figure S3: Bias component plots comparing actual educational attainment (ISCED) ●, raising of the school leaving age ■, and the educational attainment genetic score ▲ with non-genetic covariates. There was little evidence that the educational attainment allele score was more strongly associated with the phenotypic covariates than the raising of the school leaving age. There was some evidence that the score was associated with geographic location, but the size of these associations was modest.

Notes: Adjusted for the month of birth, sex, and the ten principal components of population stratification. Confidence intervals allow for clustering by month of birth. Sample weighted to adjust for under-sampling of less educated. ROSLA=Raising of the School Leaving Age.

Supplementary Figure S4: Bias component plots comparing educational attainment (ISCED) ●, raising of the school leaving age ■, and the educational attainment genetic score ▲ with genetic covariates. There was little evidence that the educational allele score was more strongly associated with the genetic confounders than the raising of the school leaving age.

Notes: Adjusted for the month of birth, sex, and the ten principal components of population stratification. Confidence intervals allow for clustering by month of birth. Sample weighted to adjust for under-sampling of less educated. ROSLA=Raising of the School Leaving Age.

Supplementary Figure S5: The effect of one additional year of schooling on morbidity, mortality and socioeconomic outcomes estimated using the educational attainment genetic score with and without weighting for under-sampling of less educated ▲ and ▲ respectively. The weighting did not affect the estimates.

Notes: Adjusted for month and year of birth, sex, and the ten principal components of population stratification. Confidence intervals allow for clustering by month of birth.

Supplementary Figure S6: The association of morbidity, mortality and socioeconomic outcomes and the educational attainment genetic score ▲ (the “reduced form”). These estimates are consistent with the main analyses presented in Figure 3.

Notes: Adjusted for month and year of birth, sex, and the ten principal components of population stratification. Confidence intervals allow for clustering by month of birth. Sample weighted to adjust for under-sampling of less educated.

Supplementary Figure S7: The effect of one additional year of schooling on morbidity, mortality and socioeconomic outcomes estimated using the educational attainment genetic score with and without adjusting for the sex, month and year of birth and principal components of population stratification ▲ and ▲ respectively. The Mendelian randomization estimates were robust.

Notes: Confidence intervals allowing for clustering by month of birth reported. Sample weighted to adjust for under-sampling of less educated.

Supplementary Figure S8: Fully adjusted results. The effect of one additional year of schooling on morbidity, mortality and socioeconomic outcomes estimated using the educational attainment genetic score with and without additionally adjusting for breastfeeding, mother smoked during pregnancy, birth weight, birth location and deprivation (easting, northing, and distance to London) ▲ and ▲ respectively. The fully adjusted estimates were comparable after including additional covariates.

Notes: Confidence intervals clustered by month of birth reported. Sample weighted to adjust for under sampling of less educated. All results adjust for month and year of birth, sex, and the ten principal components of population stratification.

Supplementary Figure S9: The effect of one additional year of schooling on morbidity, mortality and socioeconomic outcomes, estimated using the educational attainment GWAS genome-wide significant SNPs using inverse variance weighted, MR-Egger regression, weighted median and weighted modal estimators.

Notes: Confidence intervals clustered by month of birth reported. The sample was weighted to adjust for the under-sampling of less educated. All results adjust for month and year of birth, sex, and the ten principal components of population stratification. I^2^_gx_=0.21, this suggests that MR-Egger may be biased towards the null, as there is only modest variation in the SNP-educational attainment associations.

Supplementary Figure S10: The effect of education estimated allowing for a family fixed effects restricted to UK Biobank participants with at least one sibling. Min N= 7,053, max N=31,708. The within-families analyses are far less precise than the analyses using unrelated individuals. This is because the sample size is far smaller and because these estimates use the half of genetic variation that occurs within families.

Notes: Confidence intervals clustered by month of birth reported. The sample was restricted to a sample of siblings. For each outcome, analysis was restricted to families with at least two siblings with non-missing outcome values and education.

Supplementary Table S1: Summary of empirical papers in pubmed including the terms “Mendelian randomization” and “educational attainment” (N=81).

| **Outcome** | **References** |
| --- | --- |
| Age at Menarche | ^32^ |
| Allostatic load | ^33^ |
| ALS | ^34,35^ |
| Alzheimer’s disease | ^36–45^ |
| Asthma | ^46^ |
| Atrial fibrillation | ^47^ |
| Cannabis use | ^48^ |
| Cardiovascular disease | ^5,49–54^ |
| Childhood health conditions | ^55^ |
| Chronotype | ^56^ |
| Cleft lip/palate | ^57^ |
| COVID-19 | ^58–60^ |
| Depression | ^61^ |
| Drinking and smoking | ^62–67^ |
| Endometrial cancer | ^68^ |
| Epilepsy | ^69^ |
| Frailty | ^70^ |
| Gastrointestinal tract disorders | ^71,72^ |
| Heart failure | ^73^ |
| Hyperopia | ^74^ |
| Insomnia | ^75^ |
| Intracranial aneurysms | ^76^ |
| Leukocyte telomere length | ^77^ |
| Lung function and COPD | ^78^ |
| Menarche and first sexual intercourse | ^79^ |
| Mental disorders | ^80,81^ |
| Myopia | ^12^ |
| Neurodevelopmental disorders | ^82,83^ |
| Offspring birth weight | ^84^ |
| Oropharyngeal cancer risk | ^85^ |
| Parkinson's | ^86^ |
| Periodontitis | ^87^ |
| Physical activity | ^88^ |
| Physical and mental health | ^11,89^ |
| Posttraumatic stress disorder | ^90^ |
| Rheumatoid arthritis | ^91–93^ |
| Risky health behaviours | ^94^ |
| Shift work | ^95^ |
| Sleep apnoea | ^96^ |
| Stroke | ^97–100^ |
| Suicide attempts | ^101,102^ |
| Type 2 diabetes | ^103–106^ |
| Urological and reproductive health outcomes | ^107,108^ |
| Varicose veins | ^109^ |

### Supplementary Table S2: Traits with available GWAS, reference, and the number of SNPs which exceeded p<5e-05.

| Trait | MR-Base ID | GWAS Ref | Number of SNPs after LD pruning |
| --- | --- | --- | --- |
| **Anthropometry** | |  |  |
| Height | 89 | ^110^ | 628 |
| Body mass index | 835 | ^111^ | 214 |
| Body fat percentage | 999 | ^112^ | 72 |
|  |  |  |  |
| **Substance misuse** | |  |  |
| Cigarettes smoked per day | 961 | ^113^ | 43 |
| Ever vs never smoked | 962 | ^113^ | 59 |
| Age of smoking initiation | 964 | ^113^ | 35 |
| Alcohol dependence | 813 | ^114^ | 38 |
|  |  |  |  |
| **Childhood anthropometry** | |  |  |
| Birth weight | 1083 | ^115^ | 258 |
| Birth length | 29 | ^116^ | 52 |
| Infant head circumference | 28 | ^117^ | 3 |
| Age at menarche | 1095 | ^118^ | 273 |
|  |  |  |  |
| **Neuropsychiatric conditions** |  |  |  |
| Depressive symptoms | 1000 | ^119^ | 98 |
| Major depressive disorder | 805 |  | 36 |
| Autism | 806 | PGC | 58 |
| Schizophrenia | 22 | ^121^ | 377 |
| Alzheimer's disease | 298 | ^122^ | 96 |
| Bipolar disorder | 801 | ^123^ | 71 |
| PGC cross-disorder traits | 803 | ^124^ | 81 |
| Migraine in bipolar disorder | 1019 | ^125^ | 16 |
| Father’s age at death | 1092 | ^126^ | 101 |
| Mother’s age at death | 1093 | ^126^ | 98 |
|  |  |  |  |
| **Socioeconomic characteristics** |  |  |  |
| Agreeableness | 113 | ^127^ | 36 |
| Conscientiousness | 114 | ^127^ | 34 |
| Extraversion | 115 | ^127^ | 36 |
| Openness to experience | 117 | ^127^ | 34 |
| Neuroticism | 118 | ^127^ | 82 |
| Internalizing problems | 1029 | ^128^ | 56 |
| Subjective well being | 1009 | ^119^ | 75 |
| Chronotype | 1087 | ^129^ | 171 |
| Sleep duration | 1088 | ^129^ | 115 |
|  |  |  |  |
| **Cognition** |  |  |  |
| G speed factor | 1061 | ^130^ | 34 |
| Symbol search | 1062 | ^130^ | 31 |
| 8-choice reaction time | 1063 | ^130^ | 28 |
| 2-choice reaction time | 1064 | ^130^ | 37 |
| Inspection time | 1065 | ^130^ | 28 |
| Simple reaction time | 1066 | ^130^ | 38 |
| Digit symbol | 1067 | ^130^ | 35 |
| Childhood intelligence | 16 | ^131^ | 21 |
| Intelligence |  | ^132^ | 301 |
|  |  |  |  |
| **Metabolites and nutrition** |  |  |  |
| Omega-3 fatty acids | 855 | ^133^ | 89 |
| Omega-6 fatty acids | 856 | ^133^ | 100 |
| Omega-9 and saturated fatty acids | 857 | ^133^ | 89 |
| Linoleic acid (LA) | 893 | ^133^ | 88 |
| Mono-unsaturated fatty acids | 916 | ^133^ | 96 |
| Other polyunsaturated fatty acids than LA | 917 | ^133^ | 85 |
| Zinc | 1079 | ^134^ | 43 |

Supplementary Table S3: The estimated heterogeneity in the estimated effect of education across SNPs. Estimated using the I-squared statistic.^23^

| **Binary outcomes** |  | 95% Confidence interval | |
| --- | --- | --- | --- |
|  | I^2^ | Lower | Upper |
| Hypertension | 0.610 | 0.499 | 0.697 |
| Diabetes | 0.470 | 0.303 | 0.596 |
| Stroke | 0.238 | 0.000 | 0.433 |
| Heart attack | 0.152 | 0.000 | 0.371 |
| Depressive episode | 0.451 | 0.277 | 0.583 |
| Cancer | 0.063 | 0.000 | 0.298 |
| Died | 0.179 | 0.000 | 0.391 |
| Ever smoked | 0.726 | 0.655 | 0.782 |
| Currently smoke | 0.462 | 0.292 | 0.591 |
| Income over £18k | 0.661 | 0.567 | 0.734 |
| Income over £31k | 0.771 | 0.715 | 0.816 |
| Income over £52k | 0.822 | 0.782 | 0.855 |
| Income over £100k | 0.735 | 0.667 | 0.789 |
| **Continuous outcomes** |  |  |  |
| Grip strength (kg)* | 0.795 | 0.746 | 0.834 |
| Arterial Stiffness* | 0.088 | 0.000 | 0.322 |
| Height (cm)* | 0.887 | 0.865 | 0.906 |
| BMI (kg/m2)* | 0.857 | 0.826 | 0.882 |
| Diastolic blood pressure (mmHg)* | 0.787 | 0.736 | 0.828 |
| Systolic blood pressure (mmHg)* | 0.744 | 0.679 | 0.796 |
| Intelligence (0 to 13)* | 0.829 | 0.791 | 0.861 |
| Happiness (0 to 5 Likert)* | 0.111 | 0.000 | 0.340 |
| Alcohol consumption (1 low, 5 high)* | 0.769 | 0.712 | 0.814 |
| Hours of television viewing per day* | 0.842 | 0.807 | 0.870 |
| Moderate exercise (days/week)* | 0.690 | 0.606 | 0.755 |
| Vigorous exercise (days/week)* | 0.558 | 0.427 | 0.660 |

Supplementary Table S4: Unadjusted association of educational attainment and the outcomes.

|  |  | 95% Confidence intervals | |
| --- | --- | --- | --- |
| Outcome | Mean/risk difference | Lower | Upper |
| Hypertension | -0.015 | -0.016 | -0.014 |
| Diabetes | -0.005 | -0.005 | -0.004 |
| Stroke | -0.002 | -0.002 | -0.002 |
| Heart attack | -0.004 | -0.004 | -0.003 |
| Depressive episode | 0.008 | 0.008 | 0.009 |
| Cancer | -0.007 | -0.007 | -0.006 |
| Died | -0.002 | -0.003 | -0.002 |
| Ever smoked | -0.019 | -0.020 | -0.018 |
| Currently smoke | -0.007 | -0.008 | -0.006 |
| Income over £18k | 0.052 | 0.050 | 0.054 |
| Income over £31k | 0.065 | 0.064 | 0.066 |
| Income over £52k | 0.046 | 0.044 | 0.047 |
| Income over £100k | 0.012 | 0.011 | 0.013 |
| Grip strength (kg)* | 0.737 | 0.715 | 0.759 |
| Arterial Stiffness* | -0.098 | -0.112 | -0.083 |
| Height (cm)* | 0.619 | 0.602 | 0.636 |
| BMI (kg/m^2^)* | -0.181 | -0.189 | -0.174 |
| Diastolic blood pressure (mmHg)* | -0.077 | -0.098 | -0.056 |
| Systolic blood pressure (mmHg)* | -0.929 | -0.998 | -0.860 |
| Intelligence (0 to 13)* | 0.269 | 0.264 | 0.274 |
| Happiness (0 to 5 Likert)* | -0.008 | -0.010 | -0.006 |
| Alcohol consumption (1 low, 5 high)* | 0.082 | 0.080 | 0.085 |
| Hours of television viewing per day* | -0.187 | -0.191 | -0.184 |
| Moderate exercise (days/week)* | -0.041 | -0.045 | -0.037 |
| Vigorous exercise (days/week)* | 0.021 | 0.018 | 0.024 |

# References

1. Barcellos SH, Carvalho LS, Turley P. Education can reduce health differences related to genetic risk of obesity. *Proc Natl Acad Sci USA*. 2018 Oct 16;**115**(42):E9765–E9772.

2. Timpson NJ, Lawlor DA, Harbord RM, et al. C-reactive protein and its role in metabolic syndrome: mendelian randomisation study. *Lancet*. 2005;**366**(9501):1954–1959.

3. Chen L, Davey Smith G, Harbord RM, Lewis SJ. Alcohol Intake and Blood Pressure: A Systematic Review Implementing a Mendelian Randomization Approach. Minelli C, editor. *PLoS Medicine*. 2008 Mar 4;**5**(3):e52.

4. Domingue BW, Fletcher J, Conley D, Boardman JD. Genetic and educational assortative mating among US adults. *Proceedings of the National Academy of Sciences*. 2014 Jun 3;**111**(22):7996–8000.

5. Tillmann T, Vaucher J, Okbay A, et al. Education and coronary heart disease: mendelian randomisation study. *BMJ*. 2017 Aug 30;j3542.

6. Nguyen TT, Tchetgen EJT, Kawachi I, et al. Instrumental variable approaches to identifying the causal effect of educational attainment on dementia risk. *Annals of Epidemiology*. 2016 Jan;**26**(1):71-76.e3.

7. Nybom M. The Distribution of Lifetime Earnings Returns to College. *Journal of Labor Economics*. 2017 Oct;**35**(4):903–952.

8. Heckman J, Humphries JE, Veramendi G. Returns to Education: The Causal Effects of Education on Earnings, Health and Smoking [Internet]. Cambridge, MA: National Bureau of Economic Research; 2016 May. Report No.: w22291. Available from: http://www.nber.org/papers/w22291.pdf

9. Card D. Estimating the Return to Schooling: Progress on Some Persistent Econometric Problems. *Econometrica*. 2001 Sep;**69**(5):1127–1160.

10. Aakvik A, Salvanes KG, Vaage K. Measuring heterogeneity in the returns to education using an education reform. *European Economic Review*. 2010 May;**54**(4):483–500.

11. Davies NM, Hill WD, Anderson EL, Sanderson E, Deary IJ, Davey Smith G. Multivariable two-sample Mendelian randomization estimates of the effects of intelligence and education on health. *eLife*. 2019 Sep 17;**8**:e43990.

12. Mountjoy E, Davies NM, Plotnikov D, et al. Education and myopia: assessing the direction of causality by mendelian randomisation. *BMJ*. 2018 Jun 6;k2022.

13. Bycroft C, Freeman C, Petkova D, et al. The UK Biobank resource with deep phenotyping and genomic data. *Nature*. 2018 Oct;**562**(7726):203–209.

14. Oreopoulos P. Estimating Average and Local Average Treatment Effects of Education when Compulsory Schooling Laws Really Matter. *American Economic Review*. 2006 Feb;**96**(1):152–175.

15. Imbens GW, Angrist JD. Identification and Estimation of Local Average Treatment Effects. *Econometrica*. 1994 Mar;**62**(2):467.

16. Hemani G, Zheng J, Wade KH, et al. MR-Base: a platform for systematic causal inference across the phenome using billions of genetic associations. *bioRxiv* [Internet]. 2016 Dec 16; Available from: http://biorxiv.org/content/early/2016/12/16/078972.abstract

17. Okbay A, Beauchamp JP, Fontana MA, et al. Genome-wide association study identifies 74 loci associated with educational attainment. *Nature*. 2016 May 11;**533**(7604):539–542.

18. Burgess S, Thompson SG. Use of allele scores as instrumental variables for Mendelian randomization. *International Journal of Epidemiology*. 2013 Sep 3;**42**(4):1134–1144.

19. Jackson JW, Swanson SA. Toward a Clearer Portrayal of Confounding Bias in Instrumental Variable Applications. *Epidemiology*. 2015 Jul;**26**(4):498–504.

20. Torssander J. From Child to Parent? The Significance of Children’s Education for Their Parents’ Longevity. *Demography*. 2013 Apr;**50**(2):637–659.

21. Marioni RE, Ritchie SJ, Joshi PK, et al. Genetic variants linked to education predict longevity. *Proceedings of the National Academy of Sciences*. 2016 Nov 22;**113**(47):13366–13371.

22. Friedman EM, Mare RD. The Schooling of Offspring and the Survival of Parents. *Demography*. 2014 Aug;**51**(4):1271–1293.

23. Bowden J, Del Greco M F, Minelli C, Davey Smith G, Sheehan NA, Thompson JR. Assessing the suitability of summary data for two-sample Mendelian randomization analyses using MR-Egger regression: the role of the I2 statistic. *Int J Epidemiol*. 2016 Dec 1;**45**(6):1961–1974.

24. Bowden J, Davey Smith G, Haycock PC, Burgess S. Consistent Estimation in Mendelian Randomization with Some Invalid Instruments Using a Weighted Median Estimator. *Genetic Epidemiology*. 2016 May;**40**(4):304–314.

25. Hartwig FP, Davey Smith G, Bowden J. Robust inference in summary data Mendelian randomization via the zero modal pleiotropy assumption. *International Journal of Epidemiology*. 2017 Dec 1;**46**(6):1985–1998.

26. Greco M FD, Minelli C, Sheehan NA, Thompson JR. Detecting pleiotropy in Mendelian randomisation studies with summary data and a continuous outcome. *Statistics in Medicine*. 2015 Sep 20;**34**(21):2926–2940.

27. Martinussen T, Vansteelandt S, Tchetgen Tchetgen EJ, Zucker DM. Instrumental variables estimation of exposure effects on a time-to-event endpoint using structural cumulative survival models. *Biometrics*. 2017 Dec;**73**(4):1140–1149.

28. Kong A, Thorleifsson G, Frigge ML, et al. The nature of nurture: Effects of parental genotypes. *Science*. 2018 Jan 26;**359**(6374):424–428.

29. Howe LJ, Nivard MG, Morris TT, et al. Within-sibship genome-wide association analyses decrease bias in estimates of direct genetic effects. *Nat Genet*. 2022 May;**54**(5):581–592.

30. Howe LJ, Rasheed H, Jones PR, et al. Educational attainment, health outcomes and mortality: a within-sibship Mendelian randomization study. *International Journal of Epidemiology*. 2023 Jun 9;dyad079.

31. Brumpton B, Sanderson E, Heilbron K, et al. Avoiding dynastic, assortative mating, and population stratification biases in Mendelian randomization through within-family analyses. *Nat Commun*. 2020 Dec;**11**(1):3519.

32. Gill D, Del Greco M F, Rawson TM, et al. Age at Menarche and Time Spent in Education: A Mendelian Randomization Study. *Behav Genet*. 2017 Sep;**47**(5):480–485.

33. Ding X, Barban N, Mills MC. Educational attainment and allostatic load in later life: Evidence using genetic markers. *Prev Med*. 2019 Dec;**129**:105866.

34. Bandres-Ciga S, Noyce AJ, Hemani G, et al. Shared polygenic risk and causal inferences in amyotrophic lateral sclerosis. *Ann Neurol*. 2019 Apr;**85**(4):470–481.

35. Zhang L, Tang L, Xia K, Huang T, Fan D. Education, intelligence, and amyotrophic lateral sclerosis: A Mendelian randomization study. *Ann Clin Transl Neurol*. 2020 Sep;**7**(9):1642–1647.

36. Liu H, Hu Y, Zhang Y, et al. Mendelian randomization highlights significant difference and genetic heterogeneity in clinically diagnosed Alzheimer’s disease GWAS and self-report proxy phenotype GWAX. *Alzheimers Res Ther*. 2022 Jan 28;**14**(1):17.

37. Thorp JG, Mitchell BL, Gerring ZF, et al. Genetic evidence that the causal association of educational attainment with reduced risk of Alzheimer’s disease is driven by intelligence. *Neurobiol Aging*. 2022 Nov;**119**:127–135.

38. Anderson EL, Howe LD, Wade KH, et al. Education, intelligence and Alzheimer’s disease: evidence from a multivariable two-sample Mendelian randomization study. *Int J Epidemiol*. 2020 Aug 1;**49**(4):1163–1172.

39. Wu C, Wu L, Wang J, et al. Systematic identification of risk factors and drug repurposing options for Alzheimer’s disease. *Alzheimers Dement (N Y)*. 2021;**7**(1):e12148.

40. Wang H, Rosenthal BS, Makowski C, et al. Causal association of cognitive reserve on Alzheimer’s disease with putative sex difference. *Alzheimers Dement (Amst)*. 2021;**13**(1):e12270.

41. Østergaard SD, Mukherjee S, Sharp SJ, et al. Associations between Potentially Modifiable Risk Factors and Alzheimer Disease: A Mendelian Randomization Study. Gandy S, editor. *PLOS Medicine*. 2015 Jun 16;**12**(6):e1001841.

42. Seyedsalehi A, Warrier V, Bethlehem RAI, Perry BI, Burgess S, Murray GK. Educational attainment, structural brain reserve and Alzheimer’s disease: a Mendelian randomization analysis. *Brain*. 2023 May 2;**146**(5):2059–2074.

43. Larsson SC, Traylor M, Malik R, et al. Modifiable pathways in Alzheimer’s disease: Mendelian randomisation analysis. *BMJ*. 2017 Dec 6;**359**:j5375.

44. Raghavan NS, Vardarajan B, Mayeux R. Genomic variation in educational attainment modifies Alzheimer disease risk. *Neurol Genet*. 2019 Apr;**5**(2):e310.

45. Hu Y, Zhang Y, Zhang H, et al. Cognitive performance protects against Alzheimer’s disease independently of educational attainment and intelligence. *Mol Psychiatry*. 2022 Oct;**27**(10):4297–4306.

46. Li Y, Chen W, Tian S, Xia S, Yang B. Evaluating the Causal Association Between Educational Attainment and Asthma Using a Mendelian Randomization Design. *Front Genet*. 2021;**12**:716364.

47. Liu Y, Liu C, Liu Q. Education and Atrial Fibrillation: Mendelian Randomization Study. *Glob Heart*. 2022;**17**(1):22.

48. Chen D, Wang X, Huang T, Jia J. Genetic support of a causal relationship between cannabis use and educational attainment: a two‐sample Mendelian randomization study of European ancestry. *Addiction*. 2023 Apr;**118**(4):698–710.

49. Schunkert H, Erdmann J, Samani NJ, Deloukas P, Zeng L. Genetics of educational attainment and coronary risk in Mendelian randomization studies. *Eur Heart J*. 2020 Feb 14;**41**(7):894–895.

50. Carter AR, Gill D, Davies NM, et al. Understanding the consequences of education inequality on cardiovascular disease: mendelian randomisation study. *BMJ*. 2019 May 22;l1855.

51. Cao M, Cui B. Association of Educational Attainment With Adiposity, Type 2 Diabetes, and Coronary Artery Diseases: A Mendelian Randomization Study. *Front Public Health*. 2020 Apr 22;**8**:112.

52. Jia Y, Wang R, Guo D, et al. Contribution of metabolic risk factors and lifestyle behaviors to cardiovascular disease: A mendelian randomization study. *Nutr Metab Cardiovasc Dis*. 2022 Aug;**32**(8):1972–1981.

53. Zeng L, Ntalla I, Kessler T, et al. Genetically modulated educational attainment and coronary disease risk. *Eur Heart J*. 2019 Aug 1;**40**(29):2413–2420.

54. Li L, Pang S, Zeng L, Güldener U, Schunkert H. Genetically determined intelligence and coronary artery disease risk. *Clin Res Cardiol*. 2021 Feb;**110**(2):211–219.

55. Hughes A, Wade KH, Dickson M, et al. Common health conditions in childhood and adolescence, school absence, and educational attainment: Mendelian randomization study. *npj Sci Learn*. 2021 Dec;**6**(1):1.

56. Lane JM, Vlasac I, Anderson SG, et al. Genome-wide association analysis identifies novel loci for chronotype in 100,420 individuals from the UK Biobank. *Nat Commun*. 2016 Mar 9;**7**:10889.

57. Dardani C, Howe LJ, Mukhopadhyay N, et al. Cleft lip/palate and educational attainment: cause, consequence or correlation? A Mendelian randomization study. *International Journal of Epidemiology*. 2020 Aug 1;**49**(4):1282–1293.

58. Au Yeung SL, Wong THT, He B, Luo S, Kwok KO. Does ACE2 mediate the detrimental effect of exposures related to COVID-19 risk: A Mendelian randomization investigation. *J Med Virol*. 2023 Jan;**95**(1):e28205.

59. Yoshikawa M, Asaba K. Educational Attainment Decreases the Risk of COVID-19 Severity in the European Population: A Two-Sample Mendelian Randomization Study. *Front Public Health*. 2021 Jun 3;**9**:673451.

60. Jian Z, Wang M, Jin X, Wei X. Genetically Predicted Higher Educational Attainment Decreases the Risk of COVID-19 Susceptibility and Severity: A Mendelian Randomization Study. *Front Public Health*. 2021;**9**:731962.

61. Campbell D, Green MJ, Davies N, et al. Effects of depression on employment and social outcomes: a Mendelian randomisation study. *J Epidemiol Community Health*. 2022 Jun;**76**(6):563–571.

62. Rosoff DB, Clarke T-K, Adams MJ, et al. Educational attainment impacts drinking behaviors and risk for alcohol dependence: results from a two-sample Mendelian randomization study with ~780,000 participants. *Mol Psychiatry*. 2021 Apr;**26**(4):1119–1132.

63. Zhou T, Sun D, Li X, Ma H, Heianza Y, Qi L. Educational attainment and drinking behaviors: Mendelian randomization study in UK Biobank. *Mol Psychiatry*. 2021 Aug;**26**(8):4355–4366.

64. Sanderson E, Davey Smith G, Bowden J, Munafò MR. Mendelian randomisation analysis of the effect of educational attainment and cognitive ability on smoking behaviour. *Nat Commun*. 2019 Dec;**10**(1):2949.

65. Gage SH, Sallis HM, Lassi G, et al. Does smoking cause lower educational attainment and general cognitive ability? Triangulation of causal evidence using multiple study designs. *Psychol Med*. 2022 Jun;**52**(8):1578–1586.

66. Gage SH, Bowden J, Davey Smith G, Munafò MR. Investigating causality in associations between education and smoking: a two-sample Mendelian randomization study. *Int J Epidemiol*. 2018 Aug 1;**47**(4):1131–1140.

67. Shi X, Yuan W, Cao Q, Cui W. Education plays a crucial role in the pathway from poverty to smoking: a Mendelian randomization study. *Addiction*. 2023 Jan;**118**(1):128–139.

68. Wang Q, Wang R, Chen C, et al. Educational attainment and endometrial cancer: A Mendelian randomization study. *Front Genet*. 2022;**13**:993731.

69. Wang M, Zhang Z, Liu D, et al. Educational attainment protects against epilepsy independent of cognitive function: A Mendelian randomization study. *Epilepsia*. 2021 Jun;**62**(6):1362–1368.

70. Atkins JL, Jylhävä J, Pedersen NL, et al. A genome-wide association study of the frailty index highlights brain pathways in ageing. *Aging Cell*. 2021 Sep;**20**(9):e13459.

71. Sun Y, Cao X, Cao D, et al. Genetic estimation of correlations and causalities between multifaceted modifiable factors and gastro-oesophageal reflux disease. *Front Nutr*. 2022;**9**:1009122.

72. Adewuyi EO, O’Brien EK, Porter T, Laws SM. Relationship of Cognition and Alzheimer’s Disease with Gastrointestinal Tract Disorders: A Large-Scale Genetic Overlap and Mendelian Randomisation Analysis. *Int J Mol Sci*. 2022 Dec 19;**23**(24):16199.

73. Wang W, Wang J, Zhuang Z, et al. Assessment of causality between modifiable factors and heart failure: A Mendelian randomization analysis. *Asia Pac J Clin Nutr*. 2021 Jun;**30**(2):340–347.

74. Plotnikov D, Sheehan NA, Williams C, Atan D, Guggenheim JA, UK Biobank Eye and Vision Consortium. Hyperopia Is Not Causally Associated With a Major Deficit in Educational Attainment. *Transl Vis Sci Technol*. 2021 Oct 4;**10**(12):34.

75. Jansen PR, Watanabe K, Stringer S, et al. Genome-wide analysis of insomnia in 1,331,010 individuals identifies new risk loci and functional pathways. *Nat Genet*. 2019 Mar;**51**(3):394–403.

76. Sun X, Liu B, Chen Y, Lv L, Ye D, Mao Y. Modifiable risk factors for intracranial aneurysms: Evidence from genetic studies. *Int J Stroke*. 2022 Dec;**17**(10):1107–1113.

77. Bountziouka V, Musicha C, Allara E, et al. Modifiable traits, healthy behaviours, and leukocyte telomere length: a population-based study in UK Biobank. *Lancet Healthy Longev*. 2022 May;**3**(5):e321–e331.

78. Higbee DH, Granell R, Hemani G, Smith GD, Dodd JW. Lung function, COPD and cognitive function: a multivariable and two sample Mendelian randomization study. *BMC Pulm Med*. 2021 Jul 22;**21**(1):246.

79. Lawn RB, Sallis HM, Wootton RE, et al. The effects of age at menarche and first sexual intercourse on reproductive and behavioural outcomes: A Mendelian randomization study. *PLoS One*. 2020;**15**(6):e0234488.

80. Jones DP, Wootton RE, Gill D, et al. Mental Health as a Mediator of the Association Between Educational Inequality and Cardiovascular Disease: A Mendelian Randomization Study. *J Am Heart Assoc*. 2021 Sep 7;**10**(17):e019340.

81. Cai J, Wei Z, Chen M, et al. Socioeconomic status, individual behaviors and risk for mental disorders: A Mendelian randomization study. *Eur Psychiatry*. 2022 Apr 18;**65**(1):e28.

82. Dardani C, Riglin L, Leppert B, et al. Is genetic liability to ADHD and ASD causally linked to educational attainment? *International Journal of Epidemiology*. 2022 Jan 6;**50**(6):2011–2023.

83. Michaëlsson M, Yuan S, Melhus H, et al. The impact and causal directions for the associations between diagnosis of ADHD, socioeconomic status, and intelligence by use of a bi-directional two-sample Mendelian randomization design. *BMC Med*. 2022 Apr 11;**20**(1):106.

84. Liu Y, Jin C, Ni L-F, et al. Educational attainment and offspring birth weight: A bidirectional Mendelian randomization study. *Front Genet*. 2022;**13**:922382.

85. Gormley M, Dudding T, Kachuri L, et al. Investigating the effect of sexual behaviour on oropharyngeal cancer risk: a methodological assessment of Mendelian randomization. *BMC Med*. 2022 Jan 31;**20**(1):40.

86. Shi J, Tian J, Fan Y, et al. Intelligence, education level, and risk of Parkinson’s disease in European populations: A Mendelian randomization study. *Front Genet*. 2022;**13**:963163.

87. Baumeister S-E, Freuer D, Baurecht H, et al. Understanding the consequences of educational inequalities on periodontitis: A Mendelian randomization study. *J Clin Periodontol*. 2022 Mar;**49**(3):200–209.

88. Kari JT, Viinikainen J, Böckerman P, et al. Education leads to a more physically active lifestyle: Evidence based on Mendelian randomization. *Scand J Med Sci Sports*. 2020 Jul;**30**(7):1194–1204.

89. Hagenaars SP, Gale CR, Deary IJ, Harris SE. Cognitive ability and physical health: a Mendelian randomization study. *Sci Rep*. 2017 Jun 1;**7**(1):2651.

90. Polimanti R, Ratanatharathorn A, Maihofer AX, et al. Association of Economic Status and Educational Attainment With Posttraumatic Stress Disorder: A Mendelian Randomization Study. *JAMA Netw Open*. 2019 May 3;**2**(5):e193447.

91. Zhao SS, Holmes MV, Zheng J, Sanderson E, Carter AR. The impact of education inequality on rheumatoid arthritis risk is mediated by smoking and body mass index: Mendelian randomization study. *Rheumatology (Oxford)*. 2022 May 5;**61**(5):2167–2175.

92. Huang G, Cai J, Li W, Zhong Y, Liao W, Wu P. Causal relationship between educational attainment and the risk of rheumatoid arthritis: a Mendelian randomization study. *BMC Rheumatol*. 2021 Oct 21;**5**(1):47.

93. Bae S-C, Lee YH. Causal relationship between years of education and the occurrence of rheumatoid arthritis. *Postgrad Med J*. 2019 Jul;**95**(1125):378–381.

94. Viinikainen J, Bryson A, Böckerman P, et al. Does better education mitigate risky health behavior? A mendelian randomization study. *Econ Hum Biol*. 2022 Aug;**46**:101134.

95. Daghlas I, Richmond RC, Lane JM, et al. Selection into shift work is influenced by educational attainment and body mass index: a Mendelian randomization study in the UK Biobank. *Int J Epidemiol*. 2021 Aug 30;**50**(4):1229–1240.

96. Song L, Li H, Wang J, et al. Educational attainment could be a protective factor against obstructive sleep apnea: a study based on Mendelian randomization. *J Thorac Dis*. 2022 Jan;**14**(1):210–215.

97. Gao L, Wang K, Ni Q-B, et al. Educational Attainment and Ischemic Stroke: A Mendelian Randomization Study. *Front Genet*. 2021;**12**:794820.

98. Wang Z, Lu J, Weng W, Zhang L, Zhang J. Women’s reproductive traits and ischemic stroke: a two-sample Mendelian randomization study. *Ann Clin Transl Neurol*. 2023 Jan;**10**(1):70–83.

99. Harshfield EL, Georgakis MK, Malik R, Dichgans M, Markus HS. Modifiable Lifestyle Factors and Risk of Stroke: A Mendelian Randomization Analysis. *Stroke*. 2021 Mar;**52**(3):931–936.

100. Zhang W, Li Y, Li Y, et al. Genetically predicted higher educational attainment decreases the risk of stroke: a multivariable Mendelian randomization study. *BMC Cardiovasc Disord*. 2022 Jun 16;**22**(1):269.

101. Rosoff DB, Kaminsky ZA, McIntosh AM, Davey Smith G, Lohoff FW. Educational attainment reduces the risk of suicide attempt among individuals with and without psychiatric disorders independent of cognition: a bidirectional and multivariable Mendelian randomization study with more than 815,000 participants. *Transl Psychiatry*. 2020 Nov 9;**10**(1):388.

102. Li QS, Shabalin AA, DiBlasi E, et al. Genome-wide association study meta-analysis of suicide death and suicidal behavior. *Mol Psychiatry*. 2022 Oct 17;

103. Zhang J, Chen Z, Pärna K, Zon SKR van, Snieder H, Thio CHL. Mediators of the association between educational attainment and type 2 diabetes mellitus: a two-step multivariable Mendelian randomisation study. *Diabetologia*. 2022 Aug;**65**(8):1364–1374.

104. Liang J, Cai H, Liang G, et al. Educational attainment protects against type 2 diabetes independently of cognitive performance: a Mendelian randomization study. *Acta Diabetol*. 2021 May;**58**(5):567–574.

105. Na-Ek N, Srithong J, Aonkhum A, Boonsom S, Charoen P, Demakakos P. Educational level as a cause of type 2 diabetes mellitus: Caution from triangulation of observational and genetic evidence. *Acta Diabetol*. 2022 Jan;**59**(1):127–135.

106. Adams CD, Boutwell BB. Can increasing years of schooling reduce type 2 diabetes (T2D)?: Evidence from a Mendelian randomization of T2D and 10 of its risk factors. *Sci Rep*. 2020 Jul 31;**10**(1):12908.

107. Day FR, Helgason H, Chasman DI, et al. Physical and neurobehavioral determinants of reproductive onset and success. *Nat Genet*. 2016 Jun;**48**(6):617–623.

108. Wang M, Jian Z, Gao X, et al. Causal Associations Between Educational Attainment and 14 Urological and Reproductive Health Outcomes: A Mendelian Randomization Study. *Front Public Health*. 2021;**9**:742952.

109. Shadrina AS, Sharapov SZ, Shashkova TI, Tsepilov YA. Varicose veins of lower extremities: Insights from the first large-scale genetic study. *PLoS Genet*. 2019 Apr;**15**(4):e1008110.

110. Wood AR, Esko T, Yang J, et al. Defining the role of common variation in the genomic and biological architecture of adult human height. *Nature Genetics*. 2014 Oct 5;**46**(11):1173–1186.

111. Locke AE, Kahali B, Berndt SI, et al. Genetic studies of body mass index yield new insights for obesity biology. *Nature*. 2015 Feb 11;**518**(7538):197–206.

112. Lu Y, Day FR, Gustafsson S, et al. New loci for body fat percentage reveal link between adiposity and cardiometabolic disease risk. *Nature Communications*. 2016 Feb 1;**7**:10495.

113. Furberg H, Kim Y, Dackor J, et al. Genome-wide meta-analyses identify multiple loci associated with smoking behavior. *Nature Genetics*. 2010 May;**42**(5):441–447.

114. Olfson E, Bierut LJ. Convergence of genome-wide association and candidate gene studies for alcoholism. *Alcohol Clin Exp Res*. 2012 Dec;**36**(12):2086–2094.

115. Horikoshi M, Beaumont RN, Day FR, et al. Genome-wide associations for birth weight and correlations with adult disease. *Nature*. 2016 Oct 13;**538**(7624):248–252.

116. Valk RJP van der, Kreiner-Møller E, Kooijman MN, et al. A novel common variant in DCST2 is associated with length in early life and height in adulthood. *Hum Mol Genet*. 2015 Feb 15;**24**(4):1155–1168.

117. Taal HR, Pourcain BS, Thiering E, et al. Common variants at 12q15 and 12q24 are associated with infant head circumference. *Nat Genet*. 2012 Apr 15;**44**(5):532–538.

118. Perry JR, Day F, Elks CE, et al. Parent-of-origin-specific allelic associations among 106 genomic loci for age at menarche. *Nature*. 2014 Oct 2;**514**(7520):92–97.

119. Okbay A, Baselmans BML, De Neve J-E, et al. Genetic variants associated with subjective well-being, depressive symptoms, and neuroticism identified through genome-wide analyses. *Nat Genet*. 2016 Jun;**48**(6):624–633.

120. Major Depressive Disorder Working Group of the Psychiatric GWAS Consortium, Ripke S, Wray NR, et al. A mega-analysis of genome-wide association studies for major depressive disorder. *Mol Psychiatry*. 2013 Apr;**18**(4):497–511.

121. Schizophrenia Working Group of the Psychiatric Genomics Consortium. Biological insights from 108 schizophrenia-associated genetic loci. *Nature*. 2014 Jul 24;**511**(7510):421–427.

122. Lambert JC, Ibrahim-Verbaas CA, Harold D, et al. Meta-analysis of 74,046 individuals identifies 11 new susceptibility loci for Alzheimer’s disease. *Nat Genet*. 2013 Dec;**45**(12):1452–1458.

123. Psychiatric GWAS Consortium Bipolar Disorder Working Group. Large-scale genome-wide association analysis of bipolar disorder identifies a new susceptibility locus near ODZ4. *Nat Genet*. 2011 Sep 18;**43**(10):977–983.

124. Cross-Disorder Group of the Psychiatric Genomics Consortium. Identification of risk loci with shared effects on five major psychiatric disorders: a genome-wide analysis. *Lancet*. 2013 Apr 20;**381**(9875):1371–1379.

125. Jacobsen KK, Nievergelt CM, Zayats T, et al. Genome wide association study identifies variants in NBEA associated with migraine in bipolar disorder. *J Affect Disord*. 2015 Feb 1;**172**:453–461.

126. Pilling LC, Atkins JL, Bowman K, et al. Human longevity is influenced by many genetic variants: evidence from 75,000 UK Biobank participants. *Aging (Albany NY)*. 2016 Mar;**8**(3):547–560.

127. Moor MHM de, Costa PT, Terracciano A, et al. Meta-analysis of genome-wide association studies for personality. *Molecular Psychiatry*. 2012 Mar;**17**(3):337–349.

128. Benke KS, Nivard MG, Velders FP, et al. A genome-wide association meta-analysis of preschool internalizing problems. *J Am Acad Child Adolesc Psychiatry*. 2014 Jun;**53**(6):667-676.e7.

129. Jones SE, Tyrrell J, Wood AR, et al. Genome-Wide Association Analyses in 128,266 Individuals Identifies New Morningness and Sleep Duration Loci. *PLoS Genet*. 2016 Aug;**12**(8):e1006125.

130. Luciano M, Hansell NK, Lahti J, et al. Whole genome association scan for genetic polymorphisms influencing information processing speed. *Biol Psychol*. 2011 Mar;**86**(3):193–202.

131. Benyamin B, Pourcain B, Davis OS, et al. Childhood intelligence is heritable, highly polygenic and associated with FNBP1L. *Mol Psychiatry*. 2014 Feb;**19**(2):253–258.

132. Sniekers S, Stringer S, Watanabe K, et al. Genome-wide association meta-analysis of 78,308 individuals identifies new loci and genes influencing human intelligence. *Nature Genetics*. 2017 May 22;**49**(7):1107–1112.

133. Kettunen J, Tukiainen T, Sarin A-P, et al. Genome-wide association study identifies multiple loci influencing human serum metabolite levels. *Nature Genetics*. 2012 Jan 29;**44**(3):269–276.

134. Evans DM, Zhu G, Dy V, et al. Genome-wide association study identifies loci affecting blood copper, selenium and zinc. *Hum Mol Genet*. 2013 Oct 1;**22**(19):3998–4006.
